# An assessment of the knowledge, practices and resources during the delivery of malaria health care services among private health care practitioners: a cross section study in the Mid-Western Region of Uganda

**DOI:** 10.1186/s12913-021-06849-8

**Published:** 2021-08-10

**Authors:** Humphrey Wanzira, Daniel Tumwine, Patrick Bukoma, Alan Musiime, Juliet Biculu, Tom Ediamu, Samuel Gudoi, James K. Tibenderana, Ronald Mulebeke, Rebecca Nantanda, Jane Achan

**Affiliations:** 1 Uganda Paediatric Association, Kampala, Uganda; 2 Malaria Action Program for Districts, Malaria Consortium, Kampala, Uganda

**Keywords:** Malaria, Private health care workers

## Abstract

**Background:**

Approximately 50 % of the population in Uganda seeks health care from private facilities but there is limited data on the quality of care for malaria in these facilities. This study aimed to document the knowledge, practices and resources during the delivery of malaria care services, among private health practitioners in the Mid-Western region of Uganda, an area of moderate malaria transmission.

**Methods:**

This was a cross sectional study in which purposive sampling was used to select fifteen private-for-profit facilities from each district. An interviewer-administered questionnaire that contained both quantitative and open-ended questions was used. Information was collected on availability of treatment aides, knowledge on malaria, malaria case management, laboratory practices, malaria drugs stock and data management. We determined the proportion of health workers that adequately provided malaria case management according to national standards.

**Results:**

Of the 135 health facilities staff interviewed, 61.48 % (52.91–69.40) had access to malaria treatment protocols while 48.89 % (40.19–57.63) received malaria training. The majority of facilities, 98.52 % (94.75–99.82) had malaria diagnostic services and the most commonly available anti-malarial drug was artemether-lumefantrine, 85.19 % (78–91), followed by Quinine, 74.81 % (67–82) and intravenous artesunate, 72.59 % (64–80). Only 14.07 % (8.69–21.10) responded adequately to the acceptable cascade of malaria case management practice. Specifically, 33.33 % (25.46–41.96) responded correctly to management of a patient with a fever, 40.00 % (31.67–48.79) responded correctly to the first line treatment for uncomplicated malaria, whereas 85.19 % (78.05–90.71) responded correctly to severe malaria treatment. Only 28.83 % submitted monthly reports, where malaria data was recorded, to the national database.

**Conclusions:**

This study revealed sub-optimal malaria case management knowledge and practices at private health facilities with approximately 14 % of health care workers demonstrating correct malaria case management cascade practices. To strengthen the quality of malaria case management, it is recommended that the NMCD distributes current guidelines and tools, coupled with training; continuous mentorship and supportive supervision; provision of adequate stock of essential anti-malarials and RDTs; reinforcing communication and behavior change; and increasing support for data management at private health facilities.

## Background

Malaria remains a significant public health concern in Uganda [1] with approximately 12.4 million cases and 13,203 malaria deaths reported in 2018 alone [2], one of the highest malaria burden in the sub-Saharan Africa region. Malaria alone contributes to between 30 and 50 % of outpatient visits, 15–20 % of hospital admissions and 20 % of hospital deaths; most of these in children under 5 years and pregnant women [1]. Strengthening malaria case management is a key strategy of the Uganda National Malaria Control Division (NMCD) to reduce morbidity and mortality attributed to malaria [1]. This is also one of the objectives of the 2014–2020 Uganda Malaria Reduction Strategic Plan, which is to achieve and sustain at least 90 % of malaria cases in the public and private sectors and community level who receive prompt diagnosis and treatment according to national policy.

However, findings from the 2014 Malaria program Mid-term Review (MTR) indicated that programmatic focus has largely been on public health sector facilities [3]. This is a concern given that the private sector is an important source of health care delivery to a significant proportion of Ugandans. According to the 2016 Uganda Demographic Health Survey (UDHS), approximately 60 % of all children under five years with fever sought care and advice at a private health facility [4]. This sector is increasingly playing a significant role in the delivery of health care services, especially for out-patient care, where most malaria diagnosis and treatment is conducted. Specific to malaria treatment, a more recent study in Uganda found that the majority of antimalarial drugs were distributed through the private sector (54.3 %) as compared to the public sector (45.7 %) [5]. Whereas the NMCD recognizes the important role played by this sector, there has been limited engagement with private health facilities in activities such as training, quality assurance and support for data management [3], which may contribute to poor quality of care for malaria case management in these facilities. Excluding the private health sector during implementation of health care interventions may limit the overall access, coverage, equity and quality of delivery of such interventions leading to lower and even delayed achievement of impact targets [6–8].

Specific to quality of care, sub-optimal delivery of services reduces the effectiveness of interventions and also increases the risks for morbidity complications and mortality [9]. This observation is supported by 2016 WHO statistics which showed that of 5.6 million children under 5 years who died mostly from preventable causes, the majority of deaths were attributed poor quality of service delivery at the health facility level [10, 11]. The importance of quality of health care in services delivery and its potential impact on child survival is progressively being recognized [12–14] as an important additional component to improvement of health and well-being. Understanding areas of substandard quality of care is an important step towards the design and implementation of targeted interventions for improvement of health service delivery [15–20] in this sector.

In Uganda, recent studies have shown that the quality of malaria case management in private health facilities needs to be strengthened. For instance, a study conducted in Western Uganda that was assessing anti-malaria dispensing practices, showed that drug shops (owned by private health practitioners) were major sources of parenteral anti-malarials prescriptions, which should be reserved for cases of severe malaria [21]. In another study conducted in the Eastern region the examined the factors and likelihood of severe malaria among Uganda children showed that seeking malaria health care at a drug shop delayed care seeking among patients [22]. However, most of these studies were conducted in either one site or had a small sample size. This study provides a greater insight into the status of quality of care among private health facilities from the health care provider perspective related to treating children under five years, with malaria. It was conducted in setting of high malaria transmission, with a large sample size covering private health facilities located in several districts of Mid-Western region. The overall objective was to document the knowledge, practices and resources during the delivery of malaria care services, among private health practitioners in the Mid-Western region of Uganda.

## Methods

### Study design and setting

**Fig. 1:**
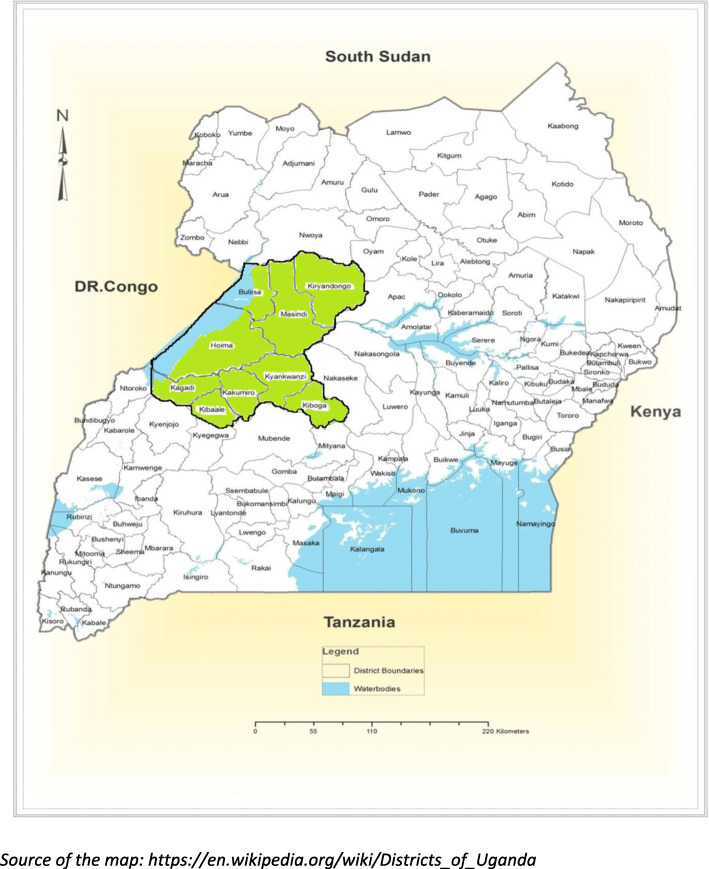
Map of Mid-Western region showing the selected districts. Source of the map: https://en.wikipedia.org/wiki/Districts_of_Uganda

This was a mixed-methods study, employing a cross sectional survey design. It was conducted in October 2018, in private-for-profit health facilities across in nine districts in the Mid-Western region of Uganda, an area with moderate to high malaria transmission [23]. The districts included Hoima, Masindi, Kiboga, Kiryandongo, Kibale, Kakumiro, Buliisa, Kagadi and Kyankwanzi districts (Fig. 1). This study was part of a larger project, assessing the quality of care of malaria health care services, in this region.

### Private health facility definition, sample size and sampling

In this study, the operational definition for a Private-For-Profit (PFP) facility was limited to those hospitals or clinics that are supervised by a medical doctor, clinical officer or nurse/midwife and utilize a business model that seeks to make a profit. From each of the nine districts, fifteen PFP facilities that fulfilled this operational definition were selected based on the assumption that 15 PFPs were representative of the district PFP coverage when considered against the average number of at least 25 PFPs found in each district [24]. There was the possibility that the number of available PFPs in the nine districts ranged from those with less than 15 and those with more PFPs. In order to maintain the 135 PFP sample size, and also ensure representativeness of the sample, the sampling of facilities at district level was conducted by probability proportion to size of the number of PFPs in each district, with districts that had less than 15 PFPs, having all facilities included, whereas those with more PFPs had more than 15 facilities sampled. Additional selection criteria for the PFPs included having a moderate to high volume of patients, employing two or more qualified medical staff and geographical spread within the district to minimize clustering and ensure representation of the district. With the assistance of the district malaria focal persons, purposive sampling was used to select these facilities giving a total of 135 PFPs across the nine (9) districts.

### Selection of survey participants

The assessment targeted health care workers that were responsible for clinical care and health facility management and included different cadres like medical and clinical officers (attained a medical diploma), nurses and nursing assistants, midwives and administrators. From each facility, one health care worker was included in the assessment. These staff members were also usually the most senior or the most knowledgeable staff in the areas of focus.

### Data Collection and study variables

An interviewer-administered pre-tested questionnaire that contained both quantitative and open-ended qualitative questions was used. This questionnaire was designed after conducting a literature and desk review (including previously used data collection tools) of the relevant areas on malaria case management in Uganda. The interviews for both data collection approaches were conducted at the same time and the data was collected by three teams each comprising of four research assistants and a supervisor. The data collectors received training that included an orientation in the study design, questionnaire and approach for data collection for both quantitative and qualitative questions. A piloting session to assess the quality of data collected for both data collection approaches was conducted, to bridge any knowledge gaps, before field work commenced. During data collection, one interviewer administered the questionnaire to one participant at a time, while completing the study questionnaire. Additional qualitative responses were noted down in a separate document and later consolidated under the qualitative study themes.

Data was collected on knowledge on malaria case management, availability of malaria treatment guidelines, malaria case management practices, laboratory practice, availability of antimalarial drugs and their stock management and reporting of malaria data (either weekly or monthly according to standard national reporting guidelines). Details of the variables assessed for during the survey are summarized in Table 1. The main outcome of interest was adequate malaria case management practices among the health facility staff. This indicator considered staff that correctly reported identifying suspected malaria cases (presenting with a fever), sending them for a confirmatory malaria test and prescribing an artemisinin based combination therapy (ACT) for uncomplicated malaria or intravenous artesunate for complicated malaria.

**Table 1:** Study variables and definitions

Assessment category	Assessment criteria/definition	Data collection
Malaria knowledge and availability of malaria treatment guidelines	• Availability and use of the following malaria treatment guidelines were documented: Integrated Malaria Management (IMM) guidelines, Malaria in Pregnancy guidelines, National treatment guidelines on treatment of severe and complicated malaria and Malaria Treatment Algorithms (2011). • Source of malaria information • Training of staff on malaria case management	Quantitative and Qualitative approaches
Malaria case management practice assessment based on the Integrated Management of Malaria (IMM) guidelines.	• Correct malaria definition (*“Malaria as an acute febrile illness caused by infection with malaria parasites. It can range from mild to severe life-threatening disease”*). • Correct definition of uncomplicated malaria (*“Symptomatic malaria without signs of severe disease”*) • Correct definition of complicated/severe malaria *(“Severe malaria is a malaria illness that is serious enough to be an immediate threat to the life of the patient”)* • Correct management of a patient with a fever (*“All patients presenting with fever should first undergo a malaria test by Rapid Diagnostic Test (RDT) or Microscopy before receiving treatment. If a patient with fever has positive test results, then it’s a confirmed Malaria diagnosis. But if a fever patient has negative malaria test results then think about other differential diagnoses for fever other than malaria”)* • Correct first line treatment of uncomplicated malaria (*prescription of an ACT, specifically artemether-lumefantrine)* • Correct first line treatment of complicated/severe malaria (*prescription of intravenous artesunate*) • Correct referral of patients (*Referral of all patients with severe/complicated malaria to a medical hospital or health facility equipped to treat such cases”)* • Correct antenatal Intermittent Preventive Therapy during pregnancy (IPTp) (*prescription of Sulphadoxine-pyremethamine ).* • Adequate malaria case management practices *( “all malaria suspected patients, with a fever, tested for malaria and those who are confirmed to have malaria are treated as per national guidelines. Uncomplicated malaria treated with artemether-lumefantrine and complicated malaria treated with intravenous artesunate)*	Quantitative and Qualitative approaches
Laboratory practices	• Presence of laboratory services, laboratory personnel, types of malaria tests, laboratory testing protocols and training of the personnel • Skilled personnel – those that have been trained on the basic malaria diagnostic practices • Adequate space – designated space to allow for the diagnosis of malaria according to the national laboratory guidelines	Quantitative and Qualitative approaches
Anti-malarial drugs stocks and stock management	• Anti-malarials used at the facility and occurrence of stock outs	Quantitative and Qualitative approaches
Data management practices	• Having a designated data records person assigned and trained on data recording and management. • Proper patient documentation process - using the forms on which malaria is reported including the Health Management Information System (HMIS) weekly and monthly forms • Evidence of data utilization (conducting analysis and presenting data, mostly as graphs) • Reporting completeness of the weekly and monthly reports as submitted into the District Health Information System 2 (DHIS2) and centrally assessed; defined as proportion of expected reports (among all registered private health facilities) that were reported to the DHIS2.	Quantitative approach

## Data management and statistical analysis

Data collection tools were checked daily for completeness and accuracy and errors were corrected before data entry. Double data entry was done using Epidata version 3.1. with range, consistency and validity checks built in to minimize errors. Stata version 14 was used for all quantitative data analysis including a descriptive analysis of all study variables, presented as frequencies with respective proportions (and 95 % Confidence Intervals) for all categorical parameters. Results were presented in tables, graphs and text. Microsoft excel was used to analyze qualitative data from the open-ended questions and additional notes. Data were transcribed, coded and analyzed using thematic analysis. Themes were developed from pre-defined topics together with themes emerging from the data. The themes were presented in text to supplement to the quantitative findings.

## Results

### Baseline characteristics

A total of 135 private for profit health facilities (PFPs) were included from nine districts as follows; 25 PFPs from Hoima, 16 PFPs from Masindi, 15 PFPs from Kakumiro, 16 PFPs from Kiryandongo, 15 PFPs from Kakumiro, 14 PFPs from Kyankwanzi, 12 PFPs from Buliisa, 11 PFPs from Kibale and 11 PFPs from Kagadi. Almost all the selected facilities, (99.26 %, 134/135), were either clinics, medical centers or nursing homes with only one hospital included as indicated by the interviewees. Most of the facilities, 63.70 %(86/135), were in urban settings. Most of the staff interviewed, 56.30 %(76/135), had worked at the facilities for more than 12 months and the majority, 71.85 % (97/135), were either clinical officers or nurses (Table 2).

**Table 2:** Study sample baseline characteristics

Variable	Number ***N*** = 135	Percentage
Location of facility		
Rural	49	36.30
Urban	86	63.70
Interviewee gender		
Male	83	61.48
Female	52	38.52
Interviewee qualification		
Clinical officer	45	33.33
Nurse	52	38.52
Nursing assistant^a^	15	10.83
Medical officer	12	9.17
Midwife	11	8.33
Interviewee professional position		
PFP Owner	43	31.85
In-charge of facility	49	36.30
Other	43	31.85
Interviewee duration of work		
> 12 months	76	56.30
< 12 and > 1 months	51	37.78
< 1 month	8	5.93

^a^Non-clinical staff who Provide basic patient care under direction of nursing staff

### Malaria case management service provision

Table 3 presents findings on malaria service provision at facility level. Two thirds of the respondents, 66.67 % (90/135), had access to malaria treatment protocols though only 61.48 % (83/135) used then routinely. The Ministry of Health was the largest source of malaria related information at 52.49 % (71/135), followed by the media and information obtained during formal education, each at 26.67 % (36/135). Less than half of the respondents, 48.89 % (66/135) had received malaria training in the last 12 months of these, only 40.74 % (55/135) had received training specific to the malaria treatment protocols and guidelines. Most of the respondents, 82.96 % (112/135), provided a correct case definition for malaria.

**Table 3:** Assessment of malaria service delivery

Assessment area	Number ***N*** = 135	Percentage (95% CI)
**Malaria treatment policy and guidelines**		
Availability of malaria treatment protocols/guidelines	95	70.37(62.02 - 77.54)
Access to malaria treatment protocols/guidelines	90	66.67(58.19 - 74.18)
Use of malaria treatment protocols/guidelines	83	61.48(52.91 - 69.40)
Heard of malaria test and treat policy	91	67.41(58.96 - 74.86)
**Source of malaria information**		
Ministry of Health	71	52.49(43.82 - 61.25)
District health team	9	6.67(3.09 - 12.28)
Media	36	26.67(19.43 - 34.59)
Colleagues	35	25.93(18.77 - 34.17)
Information obtained during formal education	36	26.67(19.43 - 34.96)
Others (seminars and workshops)	4	2.96(0.81 - 7.41)
**Staff training and mentorship**		
Respondent received malaria related training in the last 12 months	66	48.89(40.19 - 57.63)
Training on malaria treatment protocols/guidelines	55	40.74(32.37 - 49.53)
**Staff knowledge**		
Correct malaria definition	112	82.96(75.54 - 88.88)
Correct definition of uncomplicated malaria	54	40.00(31.67 - 48.79)
Correct definition of complicated/severe malaria	115	85.19(78.05 - 90.71)
**Malaria laboratory practices**		
Presence of malaria laboratory services	133	98.52(94.75 - 99.82)
Availability of adequate space for laboratory	102	75.56(67.42 - 82.54)
Presence of skilled laboratory personnel	78	57.78(48.98 - 66.22)
Training of laboratory staff on malaria testing	54	40.00(31.67 - 48.79)
Availability of malaria laboratory testing protocols		
Available and seen	53	39.26(30.97 - 48.03)
Available and not seen	22	16.30(10.50 - 23.63)
Not available	60	44.44(35.90 - 53.24)
Types of malaria tests used		
Microscopy	12	8.89(4.68 - 15.01)
Malaria RDTs	52	38.52(30.28 - 47.28)
Both	71	52.59(43.82 - 61.25)
**Anti malaria drugs stock at facility**		
Anti-malaria drugs available in stock at the facility, on day of assessment		
Artemether-Lumefantrine tablets	15	85.19(78.05 - 90.71)
Quinine (either tablets or injections)	101	74.81(66.62 – 81.89)
Artesunate (intravenous)	98	72.59(64.25 – 79.91)
Sulphadoxine – Pyrimethanine (SP) tablets	34	25.19(18.11 – 33.38)
Dihydro –artemesinin piperaquine tablets	21	15.56(9.89 – 22.79)
Chloroquine tablets	4	2.96(1.00 – 7.41)

#### Laboratory diagnosis of malaria

Almost all the facilities had malaria laboratory services, 98.52 % (133/135), but only 57.78 % (78/135), had laboratory personnel to run these laboratories. Most facilities, 52.59 % (71/135), used both microscopy and RDTs for malaria diagnosis while those that used only RDTs were 38.52 % (52/135) and 8.89 % (12/135) used microscopy only. About 39.26 % (53/135) had a laboratory testing protocol that was seen during the assessment, while 44.44 % (60/135) had no testing protocol.

There were also challenges noted under the laboratory diagnosis of malaria, during the qualitative interviews. These included the lack of skilled laboratory personnel to conduct malaria tests, the stock out of malaria testing kits (RDTs), patients not accepting negative laboratory results and others refusing to test for malaria while insisting on taking medication without a malaria test.

#### Availability of stock of anti-malaria drugs

The most commonly available anti-malarial drug in stock on the day of assessment was Artemether-Lumefantrine (AL) at 85.19 % (115/135), followed by Quinine (oral and injectable) at 74.81 (101/135) and intravenous Artesunate at 72.59 % (98/135). However, 22.22 % (30/135) of the facilities reported a stock out of anti-malarial drugs in the 3 months prior to the assessment.

According to interviews conducted, stock-outs of anti-malarial drugs such as ACTs continue to present significant challenges for these health facilities. Most staff in these facilities resorted to using any other available anti-malarial drugs, some of which are not part of the currently recommended medications like oral quinine for first line treatment.

### Malaria case management practices

**Fig. 2:**
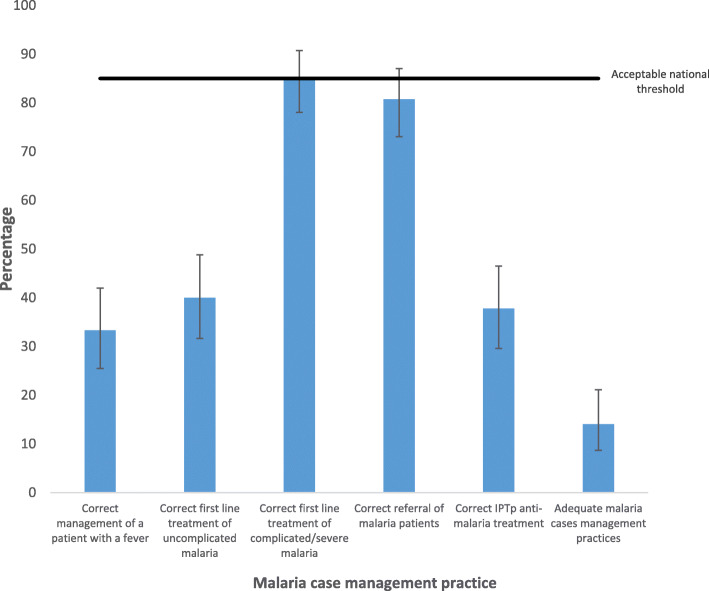
Malaria case and preventive therapy management practices in private health facilities

Figure 2 shows that only 14.07 % (19/135) of the respondents reported guideline-based malaria case management practices. Specifically, one third, 33.33 % (45/135), provided a correct response to management of a patient with a fever. Whereas only 40.00 % (54/135) provided a correct response for the first line treatment for uncomplicated malaria, a higher proportion 85.19 % (115/135), provided a correct response for treatment of complicated/severe malaria. Though 40.74 % (55/135) PFPs offered ante-natal services, 37.78 % (51/135) of the respondents knew the correct anti-malarial drug for IPTp.

A major challenge reported during the qualitative interviews was inadequate knowledge about malaria treatment among the health facility personnel that impacted case management practices, which could explain the sub-optimal guideline-based malaria case management practices. This was sometimes complicated by patients who demanded specific treatment, often contrary to treatment guidelines. In addition, as patients are charged for services at PFP facilities, those who failed to pay for a complete dose of anti-malarial treatment often had incomplete treatment.

Other challenges were related to delayed care seeking and referral practices. It was noted that some patients reported late to facilities often presenting with severe disease, others refused referrals preferring instead to continue treatment at the facilities, the latter usually occurred among those who couldn’t afford in-patient care or those who did not believe in the services offered at public health facilities. Some staff also reported difficulty in managing complicated cases such as severe anemia.

### Data management practices

A fifth of the facilities 20.74 % (28/135) had a designated data records person with only 11.85 % (16/135) trained in data recording and management. Less than half of the facilities, 48.15 % (65/135), had a proper patient documentation process while, 45.19 % (61/135), reported this information on the national HMIS forms. There was little evidence of data utilization with only 25.37 % (34/135) of facilities conducing analysis and presenting their data, mostly as graphs.

Reporting completeness of the weekly and monthly HMIS data reports was assessed among all private health facilities that were required to report into the DHIS2. The denominator used in this assessed is greater than the study sample size. This was considered as a more accurate measure for this parameter since the national DHIS2 system includes all facilities beyond the study sample.

Therefore, as shown in Table 4, only 31/163 (19 %) of the included facilities were reporting the weekly HMIS data into the DHIS and increasing to 47/163 (28 %) for those reporting the monthly HMIS data into the DHIS2.

**Table 4:** DHIS2 reporting rates of selected private health facilities

Selected district, N	HMIS malaria reporting forms	
	Weekly 033b DHIS2 reporting	Monthly 105 DHIS2 reporting
	n(%)	n(%)
Kyankwanzi, (*N* = 21)	2 (9.52%)	8 (38.10%)
Kiryandongo, (*N* = 21)	0	0
Kagadi, (*N* = 21)	3 (14.29%)	3 (14.29%)
Hoima, (*N* = 20)	8 (40.00%)	8 (40.00%)
Kiboga, (*N* = 20)	7 (35.00%)	17 (85.00%)
Kakumiro, (*N* = 18)	4 (22.22%)	4 (22.22%)
Masindi, (*N* = 16)	4 (25.00%)	4 (25.00%)
Bulisa, (*N* = 15)	0	0
Kibale, (*N* = 11)	3 (27.27%)	3 (27.27%)
**Total,*N*= 163**	**31 (19.02%)**	**47 (28.83%)**

## Discussion

The main objective of this study was to assess the knowledge, practices and resources during the delivery of malaria care services, among private health practitioners in the Mid-Western region of Uganda, an area of moderate malaria transmission. Overall, malaria case management knowledge and practices in these private facilities was sub-optimal with only 14 % of health care workers reporting that they correctly followed the malaria case management guidelines. This was defined as correctly identifying suspected malaria cases, conducting a confirmatory malaria test and prescribing an artemisinin based combination therapy (ACT) for uncomplicated malaria or intravenous artesunate for complicated malaria [1, 25]. This was lower than what was reported in the 2014 MIS that showed 36 % of children with a fever were tested for malaria before receiving treatment [23]. Whereas most health workers could correctly define a suspected malaria case, many were unable to correctly prescribe the first line treatment for uncomplicated malaria or the correct antimalarial drug for IPTp. These findings are consistent with other studies conducted in Uganda and elsewhere that showed existing gaps in appropriate malaria case management especially among children and pregnant, even in the midst of available anti-malaria drugs [5, 26, 27]. This kind of underperformance, in both private and public health facilities, could delay the achievement of the 2015–2020 UMRSP objective of attaining and sustaining prompt diagnosis and treatment for at least 90 % of malaria cases in the public and private sectors and community level, and potentially leading to higher mortality and morbidity due to malaria.

Possible reasons for this poor performance include the unavailability of the current malaria treatment protocols and guidelines for reference at the private health facilities, and lack of training, mentorship and support supervision on malaria case management. Similar findings have been previously reported by *Baily et al.* [28] and in other low and middle income countries with lack of training of heath facility staff frequently reported as a major contributor to poor performance [29]. The importance of training and supportive supervision in the improvement of the quality of care among children attending health facilities has been reported [30] and specific to malaria case management, a study conducted by *Mbonye AK et al.*, demonstrated that training improved referral of sick children seeking care at private health facilities [31].

Though the NMCD in Uganda has made significant progress in the provision of malaria case management documents and related training job aids [25], this activity did not target the private sector and largely focused on public health facilities with resulting improvements in parasitological diagnosis and treatment of confirmed malaria cases in the public facilities [1, 3] but not the private facilities. The inclusion of private health facilities as part of the strategy to strengthen health worker capacities for malaria diagnosis and treatment through regular training is one of the strategies of the 2014–2020 UMRSP which need to be implemented if the similar results are to be realized in this sector. It is essential to recognize that any planned training sessions should consider that most of the private health facilities are lower level facilities and therefore training should be tailored to the cadres running these facilities like clinical officers, enrolled nurses and laboratory assistants.

There were some positive aspects of case management noted in these PFP facilities. Unlike findings from other studies conducted in other African countries [27, 32], that showed sub-optimal understanding of malaria case management, knowledge on treatment and referral practices for severe malaria was significantly high with 85 % of health care workers reporting correct management practices. In addition, the majority of the facilities were also able to provide malaria laboratory services with RDTs mostly available. Although, almost half of the facilities lacked the requisite skilled laboratory personnel, the available facility staff were able to conduct RDT tests. Furthermore, as has been reported in other studies conducted in Uganda [5], there was availability of ACTs for treatment of uncomplicated malaria and artesunate for the treatment of complicated malaria, in most of the facilities. However, the stock out of commodities continues to be a major challenge in this setting. For instance, several studies conducted in Uganda [33, 34], reported a stock out of essential medical commodities, such as ACTs and RDTs in some facilities, an occurrence that is consistent with our study findings. Although the NMCD ensures consistent and sustainable supply and access to all malaria commodities by providing them free or highly subsidized [1], however, there is no clear strategy of how this would be implemented among private health facilities. This could partly explains the use of anti-malarials such as quinine injections, as first line treatment for both uncomplicated and complicated malaria, instead of ACTs or artesunate as recommended in the national guidelines.

The insistence of patients to be treated based on clinical diagnosis such as when they are not tested for malaria or when the test results are negative, contrary to the national guidelines [25], could be due to a lack of community awareness for correct malaria case management. This is further compounded by the practice that patients pay for the services and therefore demand to be provided a treatment of their choice irrespective of whether it is according to the national guidelines. There is evidence that focused and adequately planned behavior change communication could change this practice. Several studies have shown that community level sensitization improves health seeking behavior for malaria prevention and treatment [35, 36]. Indeed one of the strategies of the UMRSP is to strengthen malaria communication through the objective of ensuring that at least 85 % of the population practices correct malaria prevention and management measures [1]. Strategies under this objective such as; strengthening national communication framework, develop messages for different communication platforms, strengthen community behavioral change activities for malaria and improve advocacy for support for malaria control both in public and private sector should be implemented to ensure that all community members including those that seek health care in private health facilities are reached. The NMCD has package these strategies as Mass Action Against Malaria (MAAM), an approach that is currently being implemented. However, our findings demonstrate that this approach needs to be strengthened for greater coverage (through multiple communication channels) and effectiveness in message delivery.

Approximately a third of the private health facilities submitted reports with malaria related data, for monthly HMIS forms, and much less for the weekly reports (a fifth of the facilities), into the national DHIS2 system. This data unavailability and quality have been frequently reported problem in low income countries, including Uganda [37, 38]. This finding continues to undermine the capacity to make decisions about the health of the population and target resources to improve health system coverage, efficiency and quality for the country. This is especially important, in the context that a significant proportion of the population seeks care from private health facilities [4]. This demonstrates the need to for the NMCD, working with other sister health information Ministry of Health departments, to strengthen data management support among private health facilities, as is the case with public health facilities. This support should be aimed at increasing coverage of private health facilities that submit malaria data to the national DHIS2 system, distribution of national data collection registers and reporting forms to these facilities, provision of DHIS2 access for data entry and analysis, strengthened monitoring and quality assurance of data capture and transmission to the DHIS2 and the use of data for decision making.

## Study strengths and limitations

One of the strengths of this study was the large representative sample size of private health facilities covering a large region and selected from all the nine districts in the region by probability proportion to size, making this study finding generalizable to other similar settings. The use of both quantitative and qualitative approaches allowed for data triangulation and better understanding of the context to explain the quantitative information. One major weaknesses of this study is the possibility of reporting bias from the respondents who may have reported what they deemed as appropriate instead of what was accurate. However, this was minimized by data triangulation from both the quantitative and qualitative approaches. Additionally, it is acknowledged that the approach to combine both the qualitative and quantitative data collection procedures could have limited the details of responses provided, especially for the qualitative approach. However, the included questions were focused on specific themes, based on literature review, that would respond to the study objectives, using the available funds.

## Conclusions

This study revealed sub-optimal malaria case management knowledge and practices at health facility with only 14 % of health facility workers describing the correct malaria case management cascade (confirmation of suspected malaria cases and treatment of only confirmed cases), which is far below the national target of 85 %. This poor performance was mainly due to inaccessibility of current malaria case management protocols and guidelines, the lack of adequate staff training and mentorship, the stock out of essential anti-malaria commodities and inadequate malaria related community level sensitization. Additionally, approximately 29 % of facilities submitted the monthly malaria data reports to the national DHIS2 database, undermining the capacity to make population level decisions on health care, that incorporate the private health facilities.

To strengthen the quality of malaria case management at private health facilities, a health facility quality improvement approach including; the provision of the most up to date guidelines and tools, coupled with training; continuous mentorship and integrated supportive supervision; and provision of adequate stock of essential anti-malarials, is recommended. The Malaria Program should also reinforce its communication and behavior change approach for greater coverage and effectiveness in message delivery. More support is also need for data management at private health facilities including increasing coverage of facilities reporting data into the DHIS2, distribution of data collection and reporting tools coupled with a comprehensive data quality monitoring and quality assurance procedures, that includes activities conducted in combination with public health facilities.

## Data Availability

The datasets used and/or analyzed during the current study are available from the corresponding author on reasonable request.

## Abbreviations

ACTs: Artemesinin-based combination therapy
CI: Confidence interval
DHIS2: District Health Information System 2
HMIS: Health Management Information System
IMM: Integrated Malaria Management
IPTp: Intermittent Preventive Therapy during pregnancy
MAAM: Mass Action Against Malaria
MIS: Malaria Indicator Survey
MTR: Mid Term Review
NMCD: National Malaria Control Division
PFPs: Private For Profit Health Facilities
RDTs: Rapid Diagnostic Test
UDHS: Uganda Demographic Health Survey
UMRSP: Uganda Malaria Reduction and Strategic Plan
WHO: World Health Organization
